# The carcinogenic consequences of the plastic pollution crisis

**DOI:** 10.1172/JCI203775

**Published:** 2026-02-16

**Authors:** Jason A. Somarelli, Jason W. Arnold, Andrew B. West

**Affiliations:** 1 Department of Medicine, Duke Cancer Institute;; 2 Department of Molecular Genetics and Microbiology; and; 3 Department of Pharmacology and Cancer Biology, Duke University Medical Center, Durham, North Carolina, USA.

The invention of synthetic plastic polymers in the early 20th century has dramatically reshaped nearly every aspect of our daily lives. From construction materials to medical devices and food packaging, plastic pervades our modern-day existence. Its broad applicability, durability, and cost-effectiveness have led to widespread adoption for countless applications. Plastic is not a single substance but is a catchall term for a group of seven broad classes of hydrocarbon polymers. The polymers’ diversity of form and function is further enhanced through the incorporation of thousands of plastic additives, which are mixed together with the polymers to alter material properties.

Plastic was initially valued for its functional diversity and durability, but these same advantageous properties have since led to a rapidly growing plastic pollution crisis. In the 1960s, the rate of plastic production began to rise and continues to accelerate, with nearly 400 million tons produced annually worldwide. Plastic is now the most ubiquitous human-made substance in the world. Plastic becomes pollution when it enters the environment, where it can exert environmental health impacts through physical or toxicological damage. An estimated 710 million metric tons of global plastic pollution is expected between 2016 and 2040 (1). Plastic pollution will become more abundant by cumulative mass than all life on Earth, with a plethora of potential negative consequences across scales of biological organization, from molecular and cellular to population and ecosystem level (2). Because of its durability, plastic will remain a persistent, multigenerational pollutant even if plastic production halted today. Broad recognition of the plastic pollution crisis began with the discovery in the 1990s of the “great Pacific garbage patch,” a large region of plastic waste accumulating within the North Pacific Subtropical Gyre. Subsequent discovery of additional accumulations of plastic waste within ocean gyres prompted widespread recognition of the growing problem (3). Initial concerns were largely focused on human-induced environmental damage to natural spaces and wildlife. Recent reports of plastic accumulating in human tissues have drawn additional attention to the matter.

## Health impacts of micro- and nanoplastic ingestion

Of particular concern is our routine ingestion of micro- and nanoplastics (MNPs), which are defined as particles <5 mm and <1 μM, respectively. MNPs are found in food, water, and indoor air, and human consumption of hundreds of thousands of individual particles leads to ingestion of cumulative masses ranging from 100 mg to more than 5 grams of plastic each year. Despite the clear increases in bioavailability associated with nanoplastics and direct detection in human brains (4), little is known about the daily number of nanoplastic particles that might be ingested, owing to the difficulty in measuring these particles with current technologies. Ingestion rate is thought to be associated with dietary habits, water sources, and airborne exposures. Nanoplastics can pass through tissues and even penetrate cell membranes or be actively engulfed. With state-of-the-art techniques such as laser direct infrared spectroscopy and pyrolysis-gas chromatography/mass spectrometry, MNPs have been discovered in multiple human tissues, including the lungs, gastrointestinal tract, blood, heart, testis, placenta, breast milk, and brain. As we have reported, accumulation of plastic has increased in recent decades, as demonstrated by the increase in nanoplastics in decedent brains from the 2000s to the 2020s (4).

The presence of MNPs in tissues is not inert and has been associated with multiple human pathologies, though disease causality is not yet clear. For example, in a landmark study in 2024, researchers reported an association between the presence of MNPs in macrophages within carotid artery plaques and a higher risk of myocardial infarction, stroke, or death (5). Similarly, our analysis of MNPs in the brain revealed higher concentrations of MNPs in the brains of individuals with dementia (4). However, MNPs may also be difficult to disconnect from lipid-associated disease changes that also drive risk and disease progression because of their lipophilic properties. Additional research is clearly needed in both surveillance and modeling to understand the consequences of MNP, especially nanoplastic exposures and accumulations.

## Plastic exposure correlates with risk of certain cancers

Of growing concern is the potential link between chronic ingestion of MNPs and the risk of certain types of cancer. Although the picture is not yet fully clear, mounting evidence from multiple fields of study indicates a need for further investigation. One clue about the carcinogenic impacts of plastic can be found among workplace exposures. Workers exposed to polyvinyl chloride microplastics have an increased risk of liver cancers, such as angiosarcomas and hepatocellular carcinomas, and those working in plastic and rubber manufacturing are at increased risk of cancers of the breast, lung, prostate, liver, stomach, and kidney (6, 7).

Another clue is at the population health level, where we are witnessing an unsettling rise in the rates of early-onset cancers in individuals aged 35–50, pointing to a potential environmental or lifestyle change (or both) associated with this group. This age range corresponds to the generations born into the “modernized” world that brought about elevated rates of plastic ingestion, and one hypothesis is that the chronic ingestion of plastic is spurring these increases in cancer risk. Consistent with this hypothesis, elevated levels of MNPs are observed in colorectal cancer specimens as compared with noncancer colon tissue (8), and feeding studies exposing fish to MNPs have shown increased rates of liver neoplasias (9). Observations are currently merely correlative, and the generation of individuals aged 35–50 in the United States experienced, in addition to accumulating plastic waste, a number of societal changes that are likely to confound interpretation, such as changes in antibiotic use and the widespread incorporation of processed foods into our diet. Large, carefully designed epidemiological studies are needed to tease apart the multiple variables that are potentially at play. However, without better technology to measure nanoplastics, which are arguably the most concerning biological form of MNPs, surveillance efforts may be incomplete. Presently, it is also not clear whether larger microplastics serve as surrogates for bioavailable nanoplastics because of a lack of knowledge of how larger microplastics fragment and degrade in the environment; resolving this would require a concerted, large-scale, multidisciplinary effort. Though daunting, the efforts needed to fully understand the population health impact of microplastics would still represent fractional activities compared with the original scale and research outputs originally expended to develop plastics in the first place.

## MNP-induced inflammation, oxidative stress, and gut microbiome alterations

**Figure 1:**
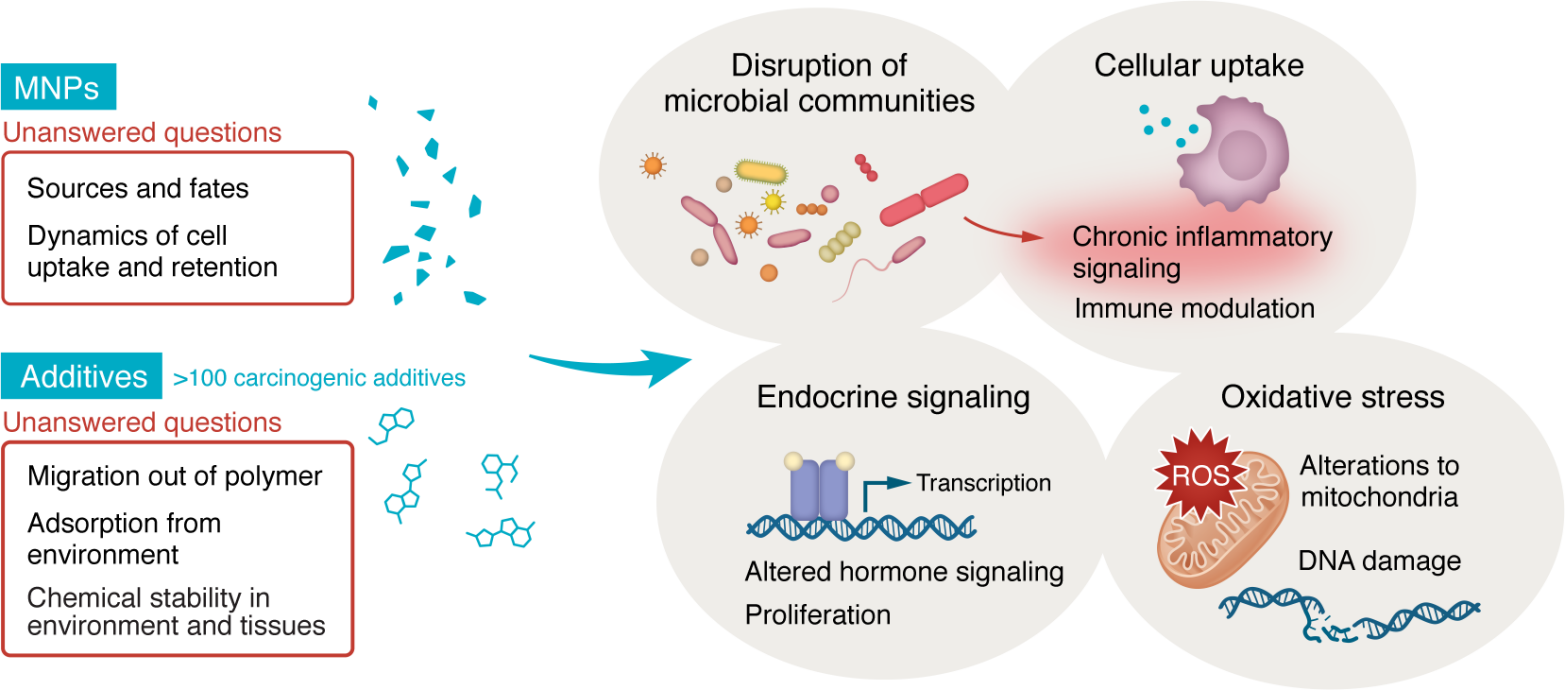
Cancer-related consequences of plastic ingestion. Both microplastics and plastic additives influence cellular processes related to cancer. Key impacts from MNPs and related additives include inflammatory signaling and immune modulation; impacts on mitochondrial energetics, oxidative stress, and DNA damage; and altered hormone signaling. Knowledge gaps remain with respect to sources and fates of MNPs, additive leaching dynamics, and the impact of material properties of MNPs on eliciting such biological effects.

One potential mechanism by which chronic MNP exposure promotes biological effects may be via a nongenotoxic, cancer cell–extrinsic mechanism. To understand how this might occur, one must first consider MNPs not as soluble toxins, but as particulate matter (PM). PM of a certain size (e.g., <2.5 μM) is a known carcinogen. But PM2.5 is not a mutagen. Rather, PM2.5 exerts cancer-promoting effects by inducing chronic inflammation, which creates a growth-permissive microenvironment for cancer. The inflammatory effects of PM2.5 are, at least partially, mediated by macrophages, which recognize and engulf foreign particles to elicit “danger” signals. The inflamed macrophages, when cocultured with small numbers of cancer cells harboring pro-proliferative EGFR mutations, promote growth of the cancer cells (10). Macrophages might recognize MNPs in a similar manner as PM2.5, engulfing plastic particles by phagocytosis and inducing pro-inflammatory signals (Figure 1). Plastic ingestion also increases gut permeability and alters composition and function of gut microbiota, which can further exacerbate inflammation. This likely induces chronic inflammation in the gut for some individuals. Combined with widespread changes in diet and gut intestinal flora in recent decades, it is possible that MNP exposure exacerbates cancer growth by creating a more cancer-permissive microenvironment. Rigorous in vivo studies are needed to further test this hypothesis, and these analyses will need to thoughtfully consider variation in plastic type; heterogeneity in shape, size, and chemistries; and the underlying diet and associated gut flora of the organism (11). In support of this hypothesis, evidence from ingestion exposure to environmentally realistic concentrations of polyethylene MNPs in mice demonstrates an increase in T cell exhaustion in colons that is mediated by IL-1β–producing macrophages (12).

In addition to pro-inflammatory signals, physical uptake of MNPs by cells and tissues can induce oxidative stress, which can lead to an increase in mutational rates and likelihood of cancer (13) (Figure 1). Examination of the exact biophysical and molecular mechanisms that underlie the generation of oxidative stress by MNPs may point to specific features of MNPs that are more harmful. For example, most laboratory studies of MNPs use commercially available spheres made of pristine polystyrene or other polymers; however, ingested MNPs in nature exhibit substantial differences from commercial MNP spheres in weathering, size distributions, roundness, material properties, and chemical composition. There is a growing need to develop standardized methods to generate and employ environmentally relevant MNPs in future studies. This work with environmentally relevant material applied at physiological concentrations will be critical to understanding how MNPs interact with disease cascades.

In addition to the interactions between MNPs and human cells and tissues, the interplay between microorganisms and MNPs in the context of cancer remains largely unexplored. Direct interactions between microorganisms and their hosts have been shown to influence inflammatory response, and compositional changes within microbial communities have been linked to cancer onset and development. MNPs may play a critical role in these dynamics. MNPs alter both the function and composition of microbial communities independent of host immune function, as has been demonstrated in environmental microbiota (14), suggesting a more drastic modulation is possible when host factors are considered, such as inflammatory activation and intestinal barrier function. Plastics themselves, especially weathered native plastics, can serve as niches to harbor bacterial communities, and ingested plastics may introduce organisms to the gut microbiota that have additional pro-inflammatory characteristics, further promoting a favorable microenvironment for tumor growth.

## Plastic additives introduce further carcinogenic potential

Impacts of plastic ingestion may be further exacerbated by the presence of plastic additives. Additives can include both intentionally and unintentionally incorporated chemicals that are noncovalently linked to the plastic. Additives can comprise a large fraction of the weight of plastic (sometimes over 50% w/v) and are chemically diverse, and additive composition differs by polymer type, product, and function (15). Studies from our team have shown that plastic additives include chemicals with known toxicological impacts, such as endocrine disruption, oxidative stress, and carcinogenesis, as well as many additives about which we know almost nothing (16, 17). Among the many additional unknowns are the fates of additives in the environment and in the body, the retention of these additives, the consequences of long-term exposure to additives at low doses, and the potential synergistic activity of complex mixtures of thousands of particles of solid polymers with unique blends of chemical additives. What we do know is that plastic can contain additives with known carcinogenic potential, pro-inflammatory chemicals, endocrine disruptors, and chemicals predicted to induce DNA damage and oxidative stress. Indeed, our analyses of well-documented plastic additives indicates over 100 known carcinogens, as classified by the International Agency for Research on Cancer, across multiple plastic products (17) (Figure 1).

Although questions of uptake and transport in the body remain, we do know that at least some of these chemicals are frequently observed in human tissues and biospecimens. Among these additives are chemicals such as bisphenol A, PFAS, formaldehyde, and heavy metal stearates. Many are known to cause oxidative stress and DNA damage, which may further exacerbate stress and inflammation induced by MNP particulate load. The impacts of chronic inflammation induced by the solid material properties of MNPs combined with oxidative stress or DNA damage brought on by plastic additives may be a particularly harmful mixture of effects. There is an urgent and growing need to conduct well-designed studies that consider such complex features of MNP exposures and do so using environmentally relevant doses, materials, and chronic exposures rather than typical high-dose, short-term exposures of commercially available MNPs.

## Meeting present and future challenges

While there is clearly more work to be done, current data suggest that long-term ingestion of MNPs is likely causing alterations to the tissue environment that are consistent with elevated risk for cancer. The complexity of the scenario from an exposure and public health perspective will present substantial challenges, and additional, rigorous studies are needed to pinpoint the environmentally relevant plastic types, sizes, shapes, additive profiles, and cumulative exposures of complex mixtures of polymers and chemicals that are most harmful. Valuable lessons should be gained from studies on polycyclic aromatic hydrocarbons, house dust, and other complex chemical mixtures. This work can be further propelled by integration of automated laboratory methodologies and artificial intelligence–guided predictive frameworks to identify the most harmful combinations of variables for further evaluation, especially plastic particles of the smallest sizes associated with heightened bioavailability. For this work to be applicable, though, we must all agree that continued preservation of environmental quality is justified to improve our long-term health and well-being. Without this shared mission, plastic pollution — and its negative impacts — will continue to grow as a major environmental and public health challenge.

## Funding support

- JAS by National Science Foundation (grant 2428209).
- JAS by Underwriters Laboratories Research Institutes.

## Version 1 02/16/2026

Electronic publication
